# Advances in clinical basic research: Performance, treatments, and mechanisms of Parkinson disease

**DOI:** 10.1002/ibra.12011

**Published:** 2021-12-23

**Authors:** Ting‐Ting Yang, Yu‐Cong Liu, Jing Li, Hui‐Chan Xu, Shun‐Lian Li, Liu‐Lin Xiong, Ting‐Hua Wang

**Affiliations:** ^1^ Department of Anesthesiology Zunyi Medical University Zunyi Guizhou China; ^2^ Department of Anesthesiology Affiliated Hospital of Zunyi Medical University Zunyi Guizhou China; ^3^ Department of Anesthesiology, Translational Neuroscience Center, Institute of Neurological Disease, West China Hospital Sichuan University Chengdu China

**Keywords:** mechanism, neurodegeneration, Parkinson disease, performance, treatment

## Abstract

The loss of neuronal in the substantia nigra of the elderly contributes to striatal damage and plays a critical part in the common forms of neurodegenerative diseases such as Parkinson disease (PD). The deficit of dopamine is one of the most familiar neuropathological features of PD as well as α‐Synuclein aggregation. The peripheral autonomic nervous system is also affected negatively during the course of the disease, although the subsistent of dyskinesias and else major motor characteristic deficits take significant role in the diagnostic methods during clinical practice, which is related to a number of non‐motor symptoms that might increase aggregate risks. Multiple pathways and mechanisms are involved in the molecular pathogenesis: α‐Synuclein, neuronal homeostasis, mitochondrial function, oxidative stress, as well as neuroinflammation. Investigations in the last few years for diagnostic biomarkers used neuroimaging, including single photon emission computed tomography  as well as cutting‐edge magnetic resonance imaging techniques, which has been presented to facilitate discrepant diagnosis. Pharmacological treatment is also important and efficient in equal measure. In addition to reliance on striatal dopamine replacement therapy, many solutions that are used for motor or nonmotor symptoms in these patients are available.

## Graphical abstract

Nonmotor symptoms can occur to varying degrees throughout the course of Parkinson disease (PD), with a prodrome that can occur for years or even decades before the onset of motor symptoms (early‐stage PD) and is characterized by the appearance of specific nonmotor symptoms (promotor PD).

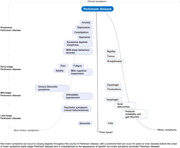

## 1 INTRODUCTION

**Figure 1:**
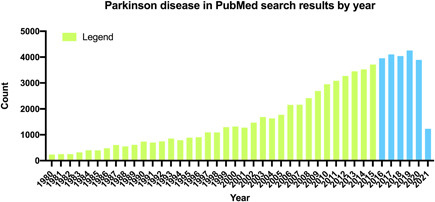
Parkinson disease in PubMed search results by year. The graph shows the number and trend of Parkinson disease searches from 1980 to 2021 (the last 5 years are in blue), indicating that research on Parkinson disease is increasing year by year [Color figure can be viewed at wileyonlinelibrary.com]

The functional capabilities of the brain decline during aging, Parkinson disease (PD) is a familiar member of age‐related brain lesions also known as tremor paralysis, which is a ubiquitous degenerative disease belonging to the nervous system of the elderly. It has characteristic motor symptoms, containing resting tremor, freezing of gait (FOG), voluntary movement, postural balance disorder, and so forth, but it also has nonmotor symptoms, containing constipation, dysarthria, sleep disorder, autonomic nerve dysfunction, and mental cognitive disorder. Nonmotor dysfunctions of PD are thought to begin long ago well‐defined motor characteristics change into evident.^1^ Dysfunction in different regional brain activities of PD increases the risk of motor deficits and nonmotor deficits, which is affected by many factors. There are different points to the mechanism of this disease, which may be related to the insufficiency of dopaminergic neurons in the substantia nigra striatum, the accumulation of neuronal misfolding protein and the increase of neuroinflammation.^2^ At present, there is no definite theory to fully explain the etiological mechanism of PD. The incidence rate of PD is high in the elderly. Simultaneously, PD is not only a great mental and psychological problem to the families but also a burden to society. Therefore, recently, new progress has been increasingly made in PD over the years (Figure 1) and it has been found that training and electrical stimulation of the deep brain can contribute to significant clinical improvement and brain plasticity. In this review, in addition to briefly summarizing the relevant clinical and basic research on PD, with a focus on disease symptoms and therapies, we will also discuss the future of the disease.

## 2 CLINICAL PERFORMANCE OF PD

### 2.1 Motor dysfunction

Gait disorders are very common in motor dysfunction of PD. Attention, stride time variability, Gait speed, and Timed Up and Go times that measured fall risk are related to gait disorders.^3^ Simieli et al. found that the PD group showed the characteristic gait deficits, indicating that condition motor planning and adaptations was initiated in the higher obstacle condition earlier than the low.^4^ Under the dual‐task condition, distraction improves the slow gait and short gait, as well as the short stride and pace used in gait training.^5^ A serious disabling symptom of this disease is freezing. The most common manifestations are gait freezing and sudden stop of an effective gait. Recent studies have shown that freezing is related to both obstacles to conflict resolution and the processing of environmental information.^6^ The cerebellar locomotor region has been found in connection with the lack of gait automaticity in individuals with FOG in this disease (freezers). The gait automaticity has been recovered and attention diversion in the freezers has been improved by the adapted resistance training with instability.^7^ A sick person with typical gait difficulty of PD are generally prone to falls and especially tend to fall at home due to FOG or balance‐related issues.^8^

### 2.2 Nonmotor dysfunction

A series of non‐motor deficiency are able to make a difference to spirit, perceive and language expression, what's more, most of these problems are totally unresponsive to drug intervention contribute to PD. And yet Altmann et al. found that aerobic exercise is one of the feasible interventions, which could not only prevent the increase of depressive symptoms but also improve some nonexercise areas, including executive dysfunction and language production.^9^ Meanwhile, executive function (EF) deficits are a core symptom of Parkinson disease dementia (PDD).^10^ Improvement of Parkinsonism patients with overactive bladder (OAB) symptoms was observed after mirabegron treatment.^11^

**Figure 2:**
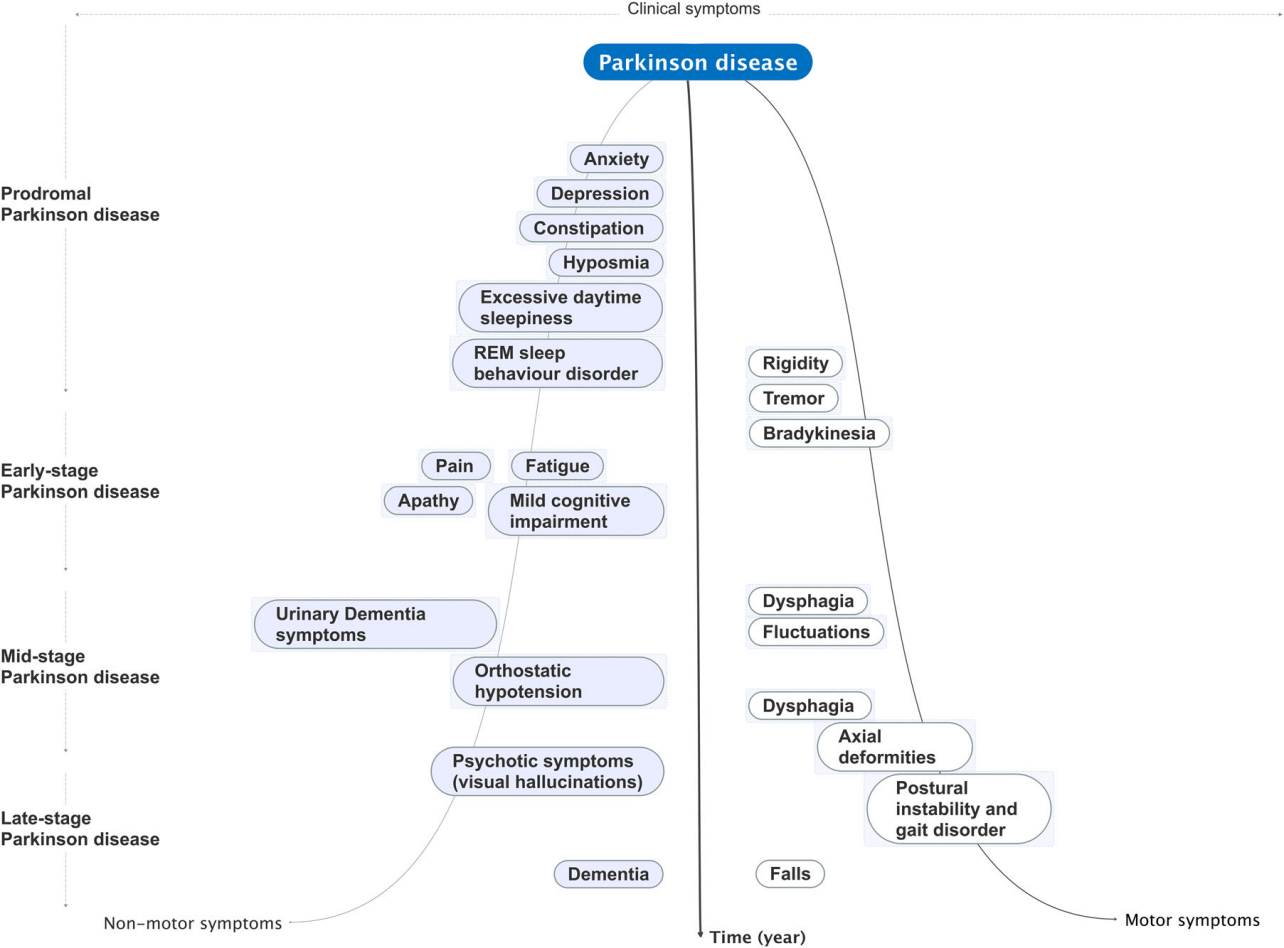
Clinical symptoms related to Parkinson disease progression. Nonmotor symptoms can occur to varying degrees throughout the course of Parkinson disease, with a prodrome that can occur for years or even decades before the onset of motor symptoms (early‐stage Parkinson disease) and is characterized by the appearance of specific nonmotor symptoms (promotor Parkinson disease). Progressive disability in Parkinson disease is driven by the combination of these nonmotor problems with the development of motor complications (mid‐stage Parkinson disease) and the evolution of motor disabilities such as postural instability. Gait problems (late‐stage Parkinson disease). REM, rapid eye movement [Color figure can be viewed at wileyonlinelibrary.com]

The motor symptoms and nonmotor symptoms of PD are shown in Figure 2.

### 2.3 Evaluation scales

Both the Unified Parkinson Disease Rating Scale (UPDRS) and the Movement Disorder Society‐sponsored revision of the Unified Parkinson Disease Rating Scale (MDS‐UPDRS) that are often used to evaluate the process and severity of this disease play an extraordinary role in diagnostic methods of this neurodegeneration disease. The severity of PD can generally be assessed by H & Y (Hoehn & Yahr) classification. Neuropsychological tests including the Tower of London task, Wisconsin Card Sorting Test (WCST), Sternberg test, Stroop and Tower of Hanoi to detect the effects of EF, depression, anxiety, mental state, manic symptoms cognitive inhibition, and quality of life.^12^, ^13^ Blood biochemistry and cerebrospinal fluid (CSF) examination performed normally.

### 2.4 Neuroimaging

Focused ultrasound (FUS) is an important and imaging‐guided adjunct to find the damage caused in deep‐brain structures, including the subthalamic nucleus (STN).^14^ What's more, magnetic resonance‐guided focused ultrasound (MRgFUS) combined with intravenous microbubble administration has been appropriate for the focal temporary opening of blood–brain barrier in patients with neurodegenerative diseases and cerebral tumors. MRgFUS may turn into a therapeutic tool to deliver medicine in nerve repair therapy.^15^ The principle of functional magnetic resonance imaging (fMRI) is to take advantage of magnetic resonance imaging for measuring the hemodynamic changes put down to the activity of neurons. It has become one of the cutting‐edge neuroimaging technologies so far.^16^, ^17^ Recent study found that the correlation between systemic oxidative stress status in patients with PD and the activity of glymphatic system is evaluated via utilizing diffusion tensor image analysis along the perivascular space.^2^ Single photon emission computed tomography, simultaneously referred to as DaTscan, has the ratify for routine application in clinical and be capable of distinguishing PD from clinical mimicry unrelated to presynaptic nigrostriatal terminal dysfunction (Table 1).^18^, ^19^

**Table 1:** Summary of neuroimaging for Parkinson disease

Category	Comment
FUS	An imaging‐guided method for creating therapeutic lesions in deep‐brain structures.^14^
MRgFUS	Applying to patients with neurodegenerative diseases and brain tumors.^15^
fMRI	A magnetic resonance imaging to measure the changes of hemodynamics caused by neuronal activity.^16^, ^17^
DTI‐ALPS	Evaluating glymphatic system activity and its relationship with systemic oxidative stress status in patients with Parkinson disease.^2^
SPECT	Distinguishing Parkinson disease from clinical mimicry unrelated to presynaptic nigrostriatal terminal dysfunction.^18^, ^19^

Abbreviations: DTI‐ALPS, diffusion tensor image analysis along the perivascular space; fMRI, functional magnetic resonance imaging; FUS, focused ultrasound; MRgFUS, magnetic resonance‐guided focused ultrasound; SPECT, single photon emission computed tomography.

### 2.5 Genetic predisposition

The majority of patients with PD normally do not have a family medical history. Fifteen percent of people who have PD but have no evident genetic pattern usually have their parents, children, or siblings with this neurodegenerative disorder.^20^ The result of twin studies shows that there is little consistency between twins when this disorder develops after 50 years of age; however, the disease progress of monozygotic twins identically before 62 years of age. It puts stress on the genetic predisposition playing a more pivotal role in early‐onset lesions than in late‐onset lesions. *PARK1* was coding for the synuclein protein. *PARK2* is associated with autosomal recessive inheritance, which encodes a protein called Parkin.^21^ It is reported that the mutation was linked to inherited Parkinson disease (*PARK5*) codes for the ubiquitin C‐terminal hydrolase L1.^22^ In the meantime, it is surprising to discover that *PARK1* mutated into *PARK6* may have a protective effect on cells.^23^ *PARK7* is not only connected to early autosomal recessive but located near the *PARK6* region on chromosome 1P36.^24^

### 2.6 CSF and blood tests

The authors found that patients with PD had heightened levels of interleukin (IL)‐1β, transforming growth factor‐β1, and IL‐6 in CSF as compared with controls. Using the systematic search, random‐effects, and meta‐analysis, they indicated that neurodegenerative diseases are associated with increased inflammatory response and the characteristic inflammatory response profile of the central nervous system of people having PD. CSF inflammatory cytokines will be used as biomarkers for many diseases in the future due to the close association between some cytokines and neurodegenerative diseases.^25^

Lawton et al. found that a range of differences between apolipoprotein A1 and C‐reactive protein levels in the subtypes of PD, and apolipoprotein A1 reduced and higher C‐reactive protein levels in severe motor disease phenotypes, poor mental health, and sleep subtypes. Not only increased C‐reactive protein but also decreased apolipoprotein A1 and Vit D had a relationship to deterioration of baseline fluctuation during daily life, using MDS‐UPDRS parts I, II, and III and Montreal Cognitive Assessment, which demonstrated that no blood biomarkers could predict exercise or nonexercise outcomes. However, baseline clinical typing might identify severe exercise or nonexercise disease phenotypes and provide biological validity.^26^

## 3 MECHANISMS LEADING TO PD

### 3.1 Neurotransmitter out‐of‐balance

Dynamic balance between dopamine that inhibitory transmitter in the striatum and acetylcholine that is an excitatory transmitter on the contrary under normal circumstances. Recent research has demonstrated that in early disease, the dramatic lack of these dopaminergic neurons is restricted to the ventrolateral substantia nigra with relatively few other midbrain dopaminergic neurons; however, it becomes more widespread in end‐stage progress of this disease.^27^ These dopaminergic neurons were largely absent even early in the disease, also suggesting that degeneration of this region begins before exercising motor symptoms.^28^, ^29^ The mechanism of selective depletion of the dopaminergic neuron is explained by the theory of transporter dysregulation.

### 3.2 α‐Synuclein aggregation

The characteristic of synucleinopathies is the aggregation of the protein α‐Synuclein, often leading to motor dysfunction as exemplified by PD.^30^ Aggregation of α‐Synuclein in oligodendrocytes is believed to be a central mechanism of the neurodegenerative process,^31^ while the severe degree of neurodegeneration and local burden of α‐Synuclein go hand in hand during the progression of PD.^28^

There are manifold factors triggering α‐Synuclein to be accumulated and aggregated by chance. For instance, impairments might be generated via the molecular pathways that degrade nature or misfolded α‐Synuclein and a relative overproduction of the protein because of potential for its misfolding and oligomerization, what's more, the accumulation of α‐Synuclein is likely to give rise to a gradual decrease in the mechanism of proteolytic defense with age.^32^, ^33^

### 3.3 Genetic factors

**Figure 3:**
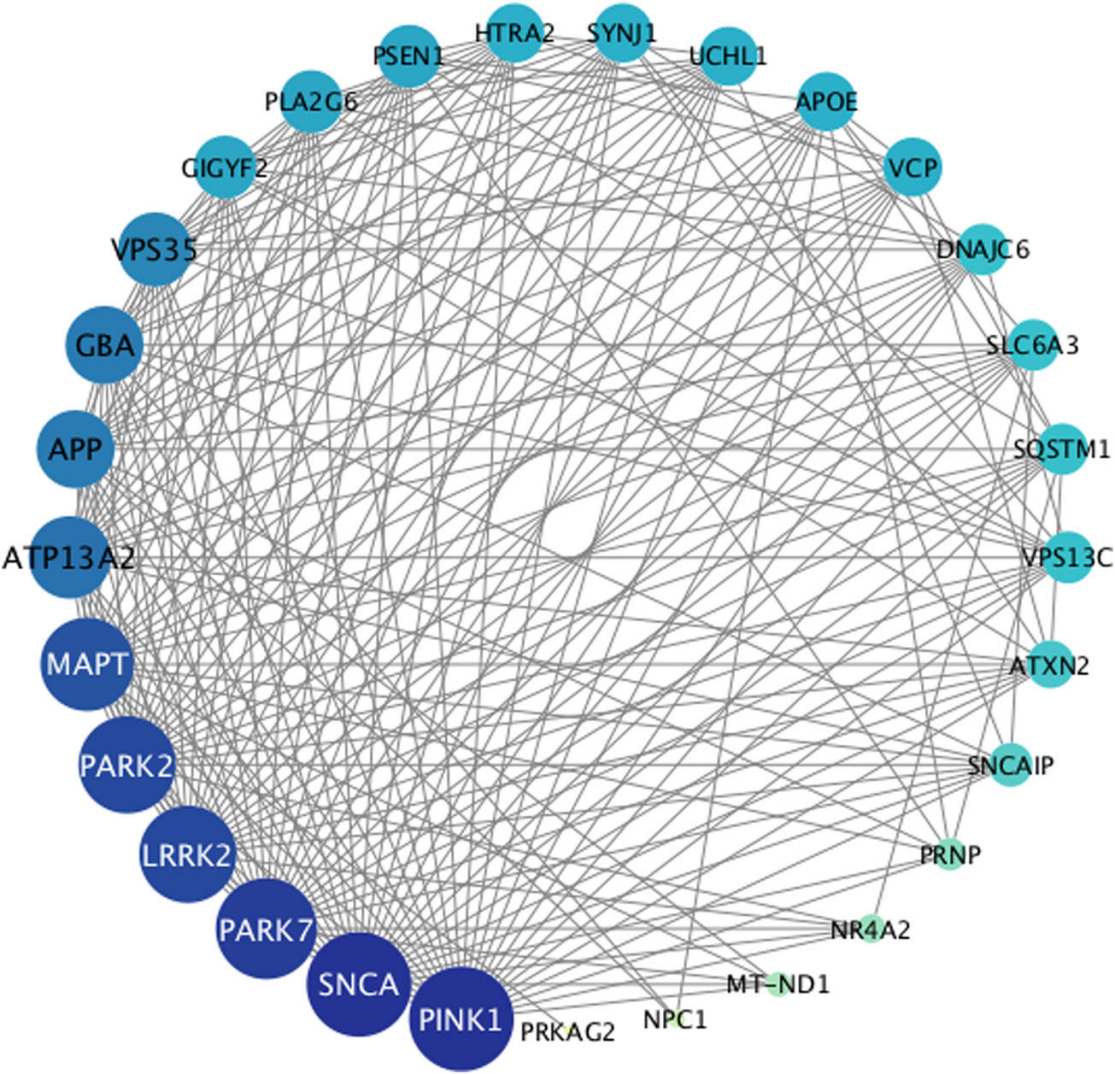
Protein–protein interaction network (PPI) related to Parkinson disease. The results showed 29 main putative targets related to PD, including *LRRK2, SNCA, PRKN, PARK7*, GBA, *PINK1, MAPT, SYNJ1, APOE, PSEN1, SNCAIP, APP, SLC6A3, VPS35, DNAJC6, PINK1‐AS, PRKAG2, HTRA2, VPS13C, UCHL1, GAA, MT‐ND1, SQSTM1, ATXN2, PLA2G6, GIGYF2, PRNP, ATP13A2, VCP, NR4A2*, and *PARK10* [Color figure can be viewed at wileyonlinelibrary.com]

Genetic factors play a significant part in the pathogenesis of PD and have attracted more and more attention recently, especially the discovery of α‐Synuclein, Parkin, and other pathogenic genes (Figure 3). Although hereditary PD accounts for only 5%–10% of cases, a range of main clues has been mentioned to the neuropathological aspect of PD. What's more, a few genes have been proved to influence sporadic disorder by large genome‐wide association studies.^34^

One of the most severe factors of risk related to PD is the mutations of the glucocerebrosidase gene named *GBA1* (*OMIM 606463*). In accordance with vitro and vivo studies, the activity of β‐glucocerebrosidase (GCase) enzyme is increased and the level of α‐Synuclein is reduced after ambroxol.^35^

*APOE ε4* has a negative association with longitudinal variations of cognition degree during the early‐stage of PD. Moreover, the increment of physical activity is beneficial to attenuate the vulnerability of *APOE ε4*, which is associated with the decrease of cognition.^36^

### 3.4 Oxidative stress

The increased evidence of it is convinced for us that oxidative stress of sick people is caused by the dysfunction of the mitochondrion.^37^ However, it is still controversial whether this phenomenon appears in the early or late stage. They found that mutations in *DJ‐1* (also known as PARK7 in humans) lead to oxidative stress of cells during the early stages of this neurodegenerative disorder.^24^, ^38^, ^39^ By knocking out *DJ‐1*, which is one of the genes related to early‐onset forms of PD and the expressions of two uncoupling proteins UCP4 (SLC25A27) and UCP5 (SLC25A14) were downregulated, calcium‐induced uncoupling and increment of oxidation of matrix proteins were compromised powerfully in the substantia nigra pars compacta (SNc) dopaminergic neurons. Moreover, glutamate can exert excitotoxic effects on nerve cells in Parkinson's pathological conditions.

### 3.5 Mitochondrial dysfunction

Both declines of mitochondrial complex I activity and increment of reactive oxygen species production are prevalent in patients with PD. Decreased mitochondrial proton pump function, decreased membrane voltage, and opening of permeability channels trigger the apoptotic process. Deletion of mitochondrial complex I not only leads to oxidative stress but also increases the susceptibility of neurons to excitotoxic death.^40^, ^41^ They pointed out that though the degree of mitochondrial α‐Synuclein often keeps low, mitochondrial protein accumulated contribute to a lack of complex I in the mitochondrion, which is a compound of the electron transport chain and oxidative stress.^42^

### 3.6 Neuroinflammation

Increasing testimonies investigate that appearance of neurodegeneration due partly to a series of collective processes have an influence on the environment. Although maybe not the initial trigger, it is probably a crucial contributor to pathogenesis.^43^

In fact, supports from experiments in Snca‐overexpressing mice point that gut microbiota plays a role in accelerating the activating progress of microglial cells as well as the disorders of motor system deficits^30^; unfortunately, it is wrong to agree that activated immune cells are merely responsible for the onset and progression in this neurodegenerative disorder. Microglia can degrade extracellular α‐Synuclein aggregates, whereas targeting α‐Synuclein immunotherapy is dependent on clearing antibody‐bound α‐Synuclein by activated immune cells.^44^

### 3.7 Norepinephrine dysfunction

Norepinephrine dysfunction is a new approach to target cognitive and behavioral deficits in PD α‐Synuclein aggregation. In addition, both imbalance and abnormal function of norepinephrine are of significance to the cognitive aspect in the early progress of PD because the locus coeruleus has degenerated. So far extensive proofs from studies of patients and animals have pointed to the role of noradrenaline in dopamine insensitivity of the PD dysexecutive syndrome, while the direct influences of norepinephrine enhancement have not been addressed to date.^45^, ^46^

### 3.8 Environmental factors

Epidemiological investigations have found regional variations in PD rates, leading to suspicions that toxic substances in the environment may be damaging brain neurons.

## 4 PHARMACOLOGICAL TREATMENTS

### 4.1 Dopaminergic therapy

Dopamine playing a critical part in making decisions has an indispensable meaning of theories and clinical implications. The fact observed by Shiner et al. showed that the activities in ventromedial prefrontal and modulated nucleus accumbent performed better than those who did not take drugs. It is indicates that dopamine could affect selective behavior that is different from mere learning by modulating the nucleus accumbent and ventromedial prefrontal cortex^47^; dopaminergic drug therapy improves decision‐making under risk, which is detectable in patients with dopamine deficiency.^48^, ^49^

Simultaneously, from the points of view of authors, dopamine could reduce the effectiveness of stimulus‐response learning and ventral activation, what's more, exogenous dopamine plays an important role in selecting accuracy associated with the enhancement DS‐BOLD signal in contrast, indicating that DS does not mediate stimulus‐response learning through feedback.^17^ Schmidt et al. suggested dopamine is crucial for shaping expectations of reward and that expectations alone may influence dopamine levels.^50^ Abnormal task‐switching costs and response repetition, which reflect impairment of activation and inhibition and persistence of selection, are increased over the course of PD regardless of reward history and are decreased after dopamine therapy. All of the effects were characterized by dopamine dependence.^46^, ^51^

The Levodopa (l‐Dopa), which is a therapeutic method for dysfunction of cognition in individuals who have PD, exacerbates impulsiveness.^52^ l‐Dopa compensates for brain damage by actively transporting and converting dopamine into the brain to serve as a substitutive therapy, which remains a mainstay in the therapeutic method to PD. Such as increasing the dose of carbidopa combined with l‐Dopa and entacapone in the treatment of volatile PD to improve the “off” time.^53^ According to the literature survey, executive‐related performance differed depending on the LD time‐to‐peak plasma concentration and task demands as well as rising LD levels were better for more difficult working memory tests.^13^ NET‐PD LS1 is a clinical trial in participants with early, mild PD on stable doses of dopaminergic therapy to compare l‐Dopa or combinational therapeutic schedule.^54^

### 4.2 Dopamine receptor agonists

There are five types of dopamine agonists that are grouped into two main classes. The two broad classes differ significantly in that when stimulated adenylyl cyclase activity changes, the D1 class promotes its elevated activity and the D2 class does not alter or decrease its activity. Dopamine receptors are distributed throughout the body. The D2 class can stimulate cardiac, renal, mesenteric arteries, and sympathetic ganglia by stimulating adenylyl cyclase to produce camp, which promotes sodium efflux, renin secretion by juxtaglomerular cells, and finally adrenal and noradrenaline release.^55^, ^56^ Free radical damage, toxic effects of excitatory amino acids, calcium overload, and mitochondrial damage is associated with the degeneration of dopaminergic neurons. Neuroprotective therapy cuts off the damage chain reaction to prevent neuronal apoptosis and deterioration. Dopamine receptor agonists also have a protective effect on neurons, which refers to the protection of neurons from various pathological insults leading to cell death that is involved in the etiology of PD, controlling the symptoms of PD by preventing or delaying the progression of PD. Dopamine agonists reduce the occurrence of complications such as “on–off” phenomenon of l‐Dopa, and can strengthen the efficacy of l‐Dopa monotherapy to delay the occurrence of complications. The authors found the combined use of low‐dose l‐Dopa and dopamine receptor agonists showed comparable efficacy to that of high‐dose l‐Dopa alone, while the incidence of side effects was significantly reduced, indicating that sublingual film provided a useful, on‐demand treatment for off episodes.^57^ Whereas it was also found by the authors that previous long‐term dopaminergic replacement may lead to tolerance to the binding of apomorphine to presynaptic autoreceptors, followed by sedation or suppression of motor activity.^58^

### 4.3 Anticholinergic drugs

Anticholinergic medicine increases the production of another neurotransmitter‐dopamine by inhibiting acetylcholine, while patients are often poorly tolerated. Cholinesterase inhibitors might beneficially influence the impairment of patients’ cognition of who also have PD and dementia, the impact likely having a connection with a substantial lack of cholinergic projections from the patients’ nucleus basalis.^59^, ^60^ Clozapine is a resultful medication aiming to symptom of psychotic aspect as well.

### 4.4 Catechol‐*O*‐methyltransferase inhibitor (COMTI)

The investigators found the phenomenon that opicapone was resultful and well‐tolerated with motor fluctuations in the participants with PD.^61^ This finding proves once again that COMTI can degrade l‐Dopa and dopamine to prolong the action time of l‐Dopa and reduce the dosage of l‐Dopa.

### 4.5 Monoamine oxidase type B inhibitor (MAOBI)

Monotherapy can be used to treat early PD and delay the progress of the disease. If combined with l‐Dopa, it can not only significantly reduce the “on–off” phenomenon and motor disorder caused by l‐Dopa but also effectively alleviate the tremor, rigidity, and motor degeneration in people with advanced PD. The researchers found the application of l‐Dopa was limited by dyskinesia and wear effects, even though it remains one of the most beneficial oral medicines for patients with PD.^62^ The primary scavenging mechanism of synaptic release of dopamine is oxidated by monoamine oxidase type B (MAOB), second only to presynaptic reuptake through dopamine transporters. The combination of evidence from patient studies suggests that the beneficial effect of safinamide, which is approved adjunct therapy to l‐Dopa for the treatment of fluctuating patients with PD may explain the reversible MAOB inhibition and regulation of glutamatergic hyperactivity.^63^

### 4.6 Noradrenaline reuptake inhibitor

Auriel et al. found that methylphenidate significantly improved attention, stride time variability, Gait speed, and Timed Up and Go times that measured fall risk but memory and visual‐spatial performance were unchangeable by observing the changes of patients before and after medication, indicating that methylphenidate enhanced mobility and decreased fall risk in patients of PD.^3^

### 4.7 Antidepressant

Depression and apathy are the main symptoms of patients with PD. Recently, selective serotonin reuptake inhibitors (SSRIs) and serotonin and norepinephrine reuptake inhibitors (SNRIs) have been developed for the therapeutic method of psychiatric symptoms of PD. SSRIs and SNRIs improve depressive symptoms of PD patients with mild to severe depressive symptoms and patients with FOG. However, their effectiveness in the treatment of apathy remains to be elucidated.^64^

### 4.8 Cholinesterase inhibitors

Rivastigmine with highly selective effects in hippocampal and cortical areas is used to increase the function of cholinergic‐releasing neurons in the brain by inhibiting acetylcholinesterase, leading to improved cognitive effects in patients with Alzheimer's disease and EF disorders in patients with PDD. The authors found that rivastigmine improved problem solving, flexibility of thinking, and planning of PDDs and affect frontal subcortical circuits.^10^

### 4.9 Atomoxetine

Atomoxetine is a neurologic agent that is used to treat attention‐deficit disorder  in the clinic principally. The authors found that atomoxetine could enhance stop‐related activation in right inferior frontal gyrus (RIFG) and increase RIFG activation and functional fronto‐striatal connectivity, which indicated that atomoxetine was able to improve response inhibition in patients with PD.^16^ More importantly, some researchers have observed that atomoxetine improves stop accuracy on stop‐signal tasks, problem‐solving function, and target sensitivity during sustained attention. And yet analysis of blood biochemistry and neuropsychological tests of 25 patients who had this neurodegenerative disorder presented that the risk of reflex impulses during gambling and information sampling could be reduced by this drug, suggesting that the drug may enhance inhibition and result in more conservative behaviors.^45^

### 4.10 Nilotinib and ambroxol

These observations in recent both vitro and vivo studies suggest that the activity of the β‐glucocerebrosidase (GCase) enzyme is increased and the level of α‐Synuclein is declined in response to ambroxol, which provide a potential method for the pathogenic pathway of ambroxol in the treatment of PD. The significant reduction of CSF GCase activity, CSF ambroxol level, GCase protein levels and α‐Synuclein concentration is aim to achieve CSF penetration and targeted binding of ambroxol.^35^

150‐mg nilotinib group showed an increase of dopamine metabolites homovanillic acid and 3,4‐dihydroxyphenylacetic acid but a decrease of α‐Synuclein oligomers and hyperphosphorylated tau levels. An increase of 3,4‐dihydroxyphenylacetic acid and hyperphosphorylated tau levels in 300‐mg nilotinib group was observed by contraries, which indicate that nilotinib showed security and detectability in the CSF and exploratory biomarkers were changed after nilotinib.^65^

### 4.11 Probiotic

Accumulating evidence suggests a link between the gut and PD.^66^ In recent 3 years, studies have shown that multistrain probiotics (hexbio) that were consumed to improve the intestinal opening frequency and the whole intestinal transmission time, and also have a beneficial effect on MDS‐UPDRS and a small number of metabolic profiles, which indicates that a variety of probiotics are effective in the drug therapies of constipation in patients with PD. It is necessary to further study the long‐term efficacy and safety of probiotics on PD, and their mechanism of action as well.^67^, ^68^, ^69^

### 4.12 Mirabegron and nabilone

Mirabegron was practical with acceptable harmful events in treating OAB symptoms in Parkinsonism's patients.^11^ The results of recent research stress the nabilone has potential efficacy for Parkinson's patients with disturbing nonmotor symptoms.^70^

### 4.13 Immunotherapy

PD01A and PD03A are two novel therapeutic vaccine candidates containing short peptides as antigenic moieties that are designed to induce a sustained antibody response that specifically targets pathogenic assemblies of α‐Synuclein.

Both PD01A and PD03A are two new‐type therapeutic vaccine candidates containing short peptides as antigenic moieties, which are intended to lead to a sustained antibody response that particularly targets the pathogenic assemblies of α‐Synuclein. The authors found that PD01‐specific antibody response of 89% of patients was developed after receiving all injections while in the PD03A‐treated group, by receiving injections of either PD01A, PD03A or placebo in those patients, titers and antibody responder rate (58%) were lower, indicating that both PD01A, which particularly aimed to the epitope of α‐Synuclein and PD03A were secure and tolerated effectively.

Both PD01A and PD03A, specifically targeted the α‐Synuclein epitope, are safe and well tolerated.^31^

Additionally, azathioprine immunosuppression is also an effective treatment for PD, and the investigators found a trial has attempted to provide evidence of mechanisms that influence disease progression by assessing the effects of azathioprine on CSF, blood, and neuroimaging parameters of immune activation in the trial population.^71^

**Figure 4:**
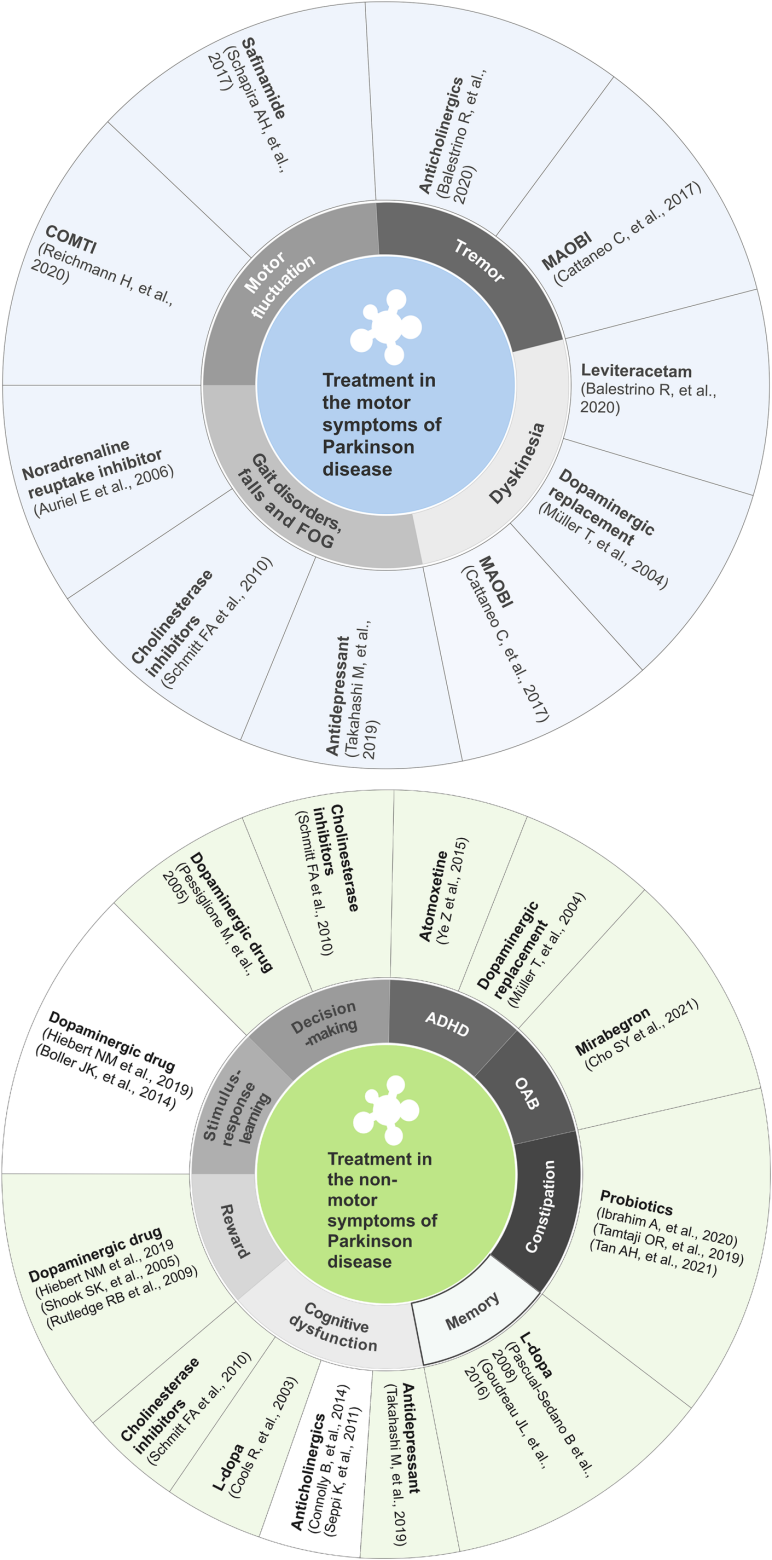
Pharmacological treatments in the symptoms of Parkinson disease. ADHD, attention‐deficit disorder; COMTI, catechol‐*O*‐methyltransferase inhibition; FOG, freezing of gait; ld/l‐Dopa, levodopa; MAOBI, monoamine oxidase type B inhibitor; OAB, overactive bladder [Color figure can be viewed at wileyonlinelibrary.com]

The summary of treatments in the symptoms is shown in Figure 4.

## 5 NONMEDICINE TREATMENT

### 5.1 Deep brain stimulation (DBS) and different brain regions related to functions

**Figure 5:**
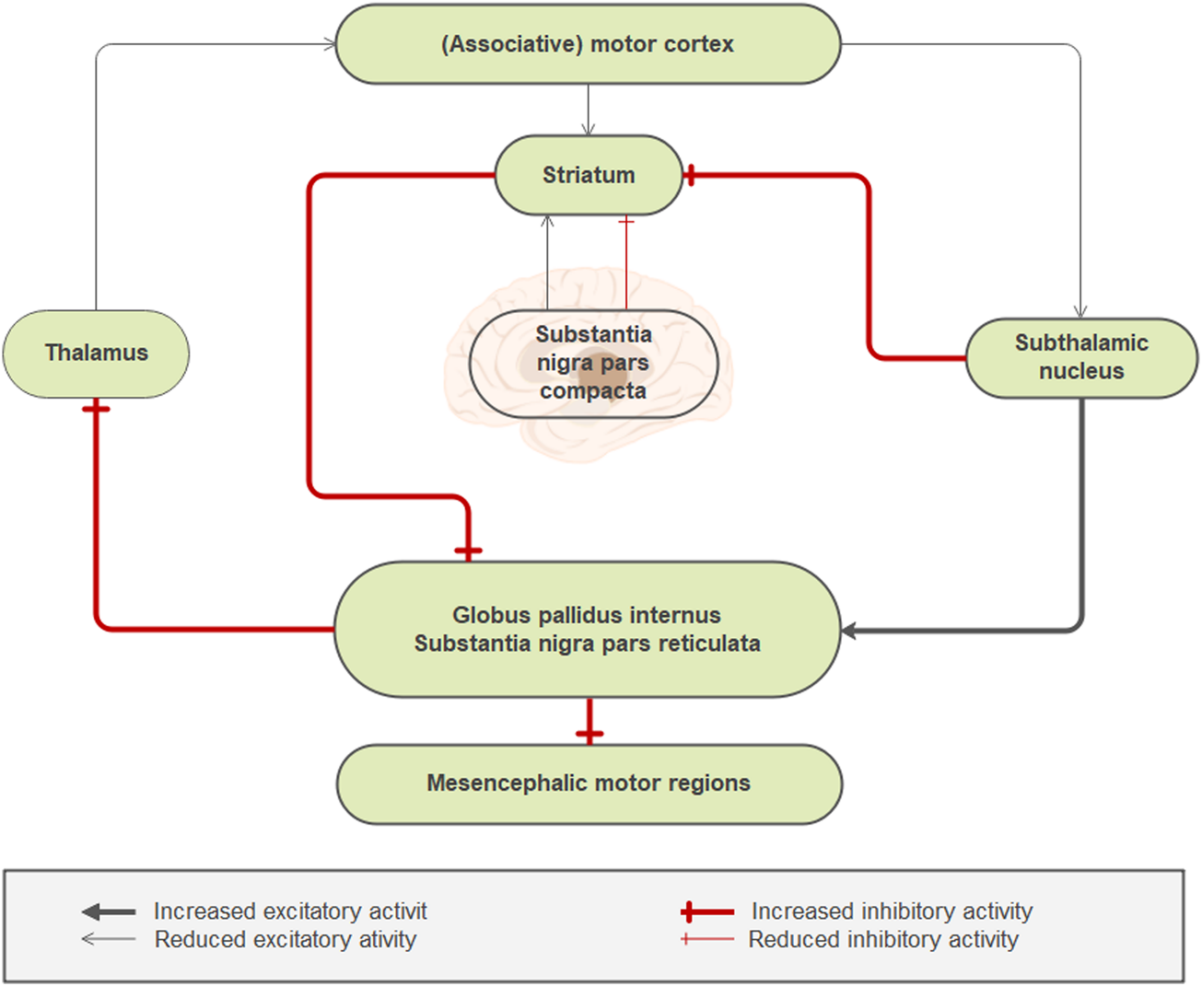
Motor cortex circuit activity changes in patients with Parkinson disease. Different functions associated with different brain regions and dopamine deficiency in the substantia nigra striatum results in the opposite effects [Color figure can be viewed at wileyonlinelibrary.com]

DBS therapy delivers electrical stimulation through localized deep brain nuclei. Stimulation of these parts can control the circuits of motor function to return to relatively normal, which is the most effective and advanced means for the treatment of functional brain diseases. DBS of the STN has a positive effect on making decisions under risk and Semantic and affective priming.^47^, ^48^ The basal ganglia circuit is associated with the recognition of the STN,^72^ which will become a novel target for DBS (Figure 5). The response inhibition can be improved via both the activation of the prefrontal cortex and the connectivity fronto‐striatum.^16^ STN is particularly thought to contribute cortico‐striatal function during decision conflict to buy time till the right decision is made.^73^ The STN as a neurosurgical target for DBS to treat the main motor feature of PD is the first preference.^14^ HF‐STN stimulation furthered PD‐associated gait disorders. To better explore this treatment, further multicenter research is necessary.^74^ Whereas bilateral STN‐DBS could lead to the verbal fluency decline selectively.^75^ Medial prefrontal cortex is broadly involved in effortful control over behavior.^73^ Only the application of the right dorsolateral prefrontal cortex (repetitive transcranial magnetic stimulation [rTMS]) to the right dorsolateral prefrontal cortex caused positive changes in the spatial planning task performance in PD, which indicates the causal engagement of the right‐sided hemispheric structures in task solving.^76^ Incidental evaluations of negative and neutral words are modulated via basal ganglia‐thalamocortical circuits in PD.^77^ The modulation of the nucleus accumbens and ventromedial prefrontal cortex by dopamine exerts a unique effect on choice behavior is different from pure learning.^47^ Dorsal striatum does not intercede with feedback‐based, stimulus‐response learning.^17^ Fronto‐striatal circuits play a role in mechanisms of working memory and attention and contributes to rule‐guided behavior.^78^, ^79^ Problem solving, flexibility of thinking, and planning of patients with PDD were associated with frontal subcortical circuits.^10^ The basal level of dopamine function in underlying cortico‐striatal circuitry is the determining factor in whether dopaminergic medication is useful or harmful to cognitive performance.^52^ Attention and motor function may be related to pallidus‐thalamic‐cortical neural circuitry.^80^

### 5.2 rTMS, pallidotomy, and subthalamotomy

Transcranial magnetic stimulation (TMS) acts on the brain through the magnetic field generated by the current in the stimulation coil, resulting in induced current in the brain, resulting in changes in cerebral blood flow, brain metabolism, brain function, and so forth. rTMS is one TMS, which not only modulates neural function during stimulation but also has a significant modulation effect after the stimulation. rTMS has been widely used in the treatment of psychoneurosis. rTMS of the dorsolateral prefrontal cortex has improvements in sentiment, but not in movement disorders and has the characteristics of noninvasive, painless, and safe.^76^ Unilateral pallidotomy with no global adverse cognitive consequences^75^ lead to immediate selective executive impairment and enhances attention and motor function,^81^ which is related to pallido‐thalamic‐cortical neural circuitry.^80^ However, there are still some patients with poor postoperative efficacy, and further research is still needed. Motor functions in selected patients with PD and asymmetric signs are enhanced by FUS subthalamotomy in one hemisphere.^14^

### 5.3 Specific exercise

Aerobic exercise not only enhances neural processing and nonmotor pathways outside those impacted by medication but also improved peak and submaximal cardiopulmonary function of patients with PD.^82^, ^83^ Karate may be an unexpected training that can improve motor disorders in Parkinson's patients, which even build up the living quality, and well‐being improved significantly for the patients with mild and moderate.^84^ Multisystem balance training reduced injurious fall risk up and lowered balance‐related fall risks in the patients of PDs.^85^ Moreover, Thaut et al. gave two different groups of interference that were finished in 30 min of home‐based gait training with metronome click‐embedded music every day, indicating that rhythmic auditory stimulation significantly reduced falls during PD and improved key gait parameters involving the velocity and the length of stride.^53^ Strand et al. found that the sample in the 30‐s STS, SMBT, Mini‐BESTest, upper‐body strength, and lower‐body strength were improved, indicating that periodized resistance training was beneficial to functional capacity, balance, and muscular strength.^86^

### 5.4 Novel training

Customized interactive video game‐based (IVGB) training is able to improve balance, postural stability, and confidence of fall prevention in old people with mild‐to‐moderate PD, even though the IVGB exercise cannot significantly affect living quality in individuals who have PD.^87^ HiCommunication program, which regards voice, articulation, word‐finding, and memory as core target areas, utilizes the completion of speech recording and dysarthria test to evaluate the possible changes in speech level and intelligibility of Parkinson's patients. HiCommunication as a novel speech treatment has positive associations with communication function in individuals having PD.^88^ Compared with traditional paper‐and‐pencil cognitive training (PCT) by means of Core software, computer‐based cognitive training has been proved to be effective in patients having PD and mild cognitive impairment. Further follow‐up evaluations are under way to verify the retention of gain and the potential ability of the tool to delay the conversion to PD dementia (Table 2).^89^

**Table 2:** Summary of nonmedicine treatment

Category	Comments
DBS	Delivering electrical stimulation through localized deep brain nuclei, it is the most effective and advanced means for the treatment of functional brain diseases.^10^, ^14^, ^16^, ^47^, ^48^, ^52^, ^80^, ^85^
rTMS	Having improvements in sentiment, but not in movement disorders.^76^
Pallidotomy	Enhancing attention and motor function, which is related to pallido‐thalamic‐cortical neural circuitry.^75^
Subthalamotomy	Improving motor function in patients with PD.^14^
Aerobic exercise	Enhancing neural processing and nonmotor pathways outside those impacted by medication and cardiopulmonary function.^82^, ^83^
Karate	Improving quality of life and well‐being improved significantly for patients with PD.^84^
RAS	Reducing the possibility of falls in patients with PD.^53^
Periodized resistance training	Enhancing functional ability, balance, and muscle strength.^86^
Customized IVGB training	Improving balance, postural stability, and confidence in preventing falls in older adults.^87^
Hicommunication	A novel speech treatment is positively associated with communication function.^88^
CCT	CCT was effective in PD‐MCI patients compared with traditional PCT.^89^

Abbeviations: CCT, computer‐based cognitive training; DBS, deep brain stimulation; IVGB, interactive video game‐based; PCT, paper‐and‐pencil cognitive training; PD, Parkinson's disease; RAS, rhythmic auditory stimulation; rTMS, repetitive transcranial magnetic stimulation.

### 5.5 Gene therapy and transplantation

With the development of new protocols for the nigra A9 dopaminergic cells from stem cell sources, renewed optimism about cell replacement is generated. However, they raised further questions after posthumous evidence of Louie's body pathology was first reported in 2008 in patients who received human fetal ventral mesencephalon grafts during the transplant. This finding is confirmed in a few follow‐up studies as well and there is evidence that the pathological burden increases with time.^90^ These incredible observations have led to new theories regarding the pathogenesis and pathological transmission of PD, with the emerging hypothesis that α‐Synuclein could function in a prion‐like manner,^91^ but for reasons that remain unclear, this phenomenon only affects a small percentage of transplanted dopaminergic cells, with no more than 10%–15% of cells having Lewy body lesions after transplantation.^92^

## 6 CONCLUSIONS AND PROSPECTS

**Figure 6:**
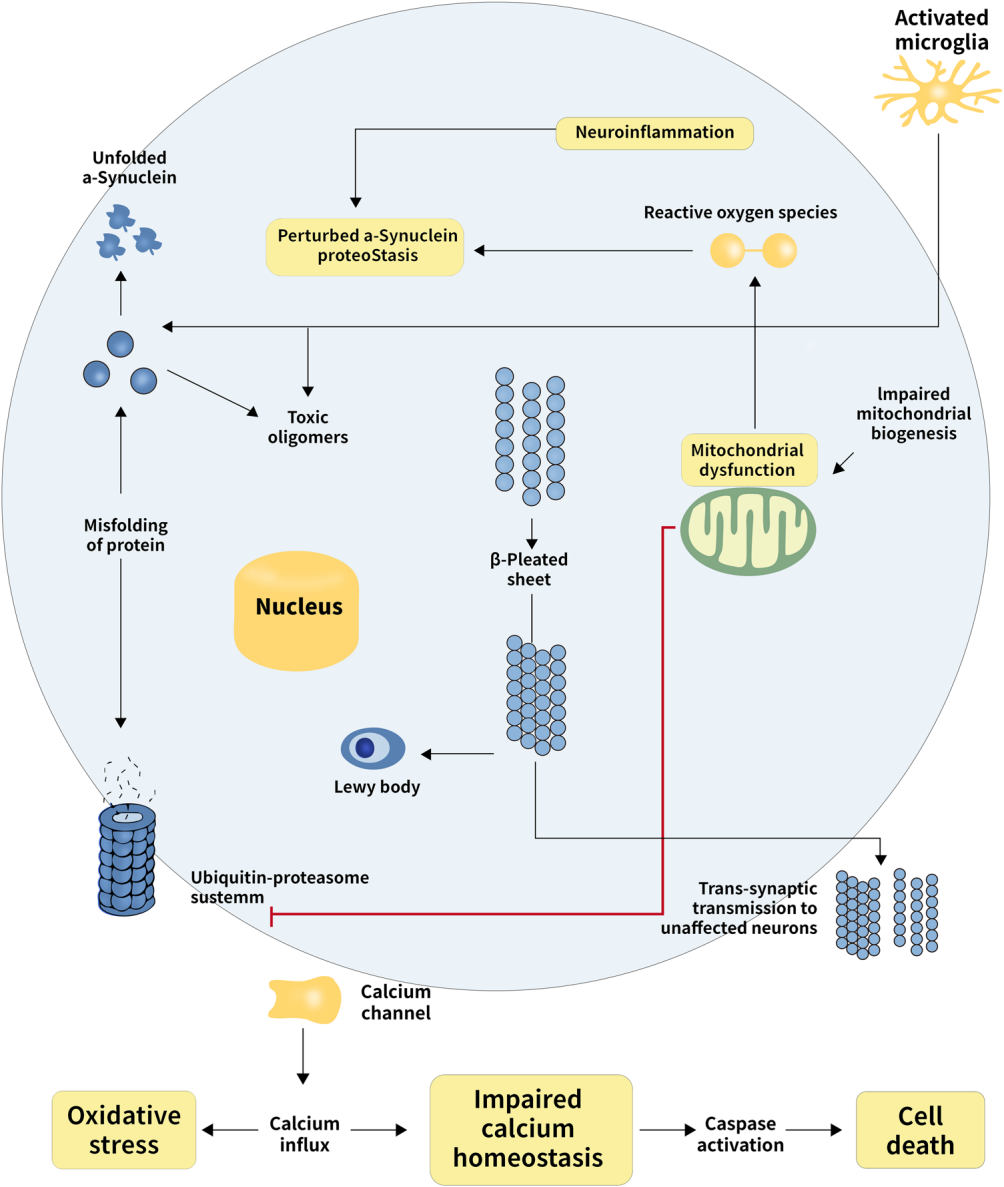
Molecular mechanisms referred to Parkinson disease. The interactions between major molecular referred to the pathogenesis of Parkinson disease are depicted by the schematic diagram in detail [Color figure can be viewed at wileyonlinelibrary.com]

Heredity is the basis of PD, the environment is the inducing factor, and immune deficiency, oxidative stress, and mitochondrial dysfunction are the intermediate processes, which eventually lead to the apoptosis of dopaminergic nerve cells in the substantia nigra striatum. Therefore, a major challenge in Parkinson's research is environmental (such as pollutants and diet, exercise, and smoking) factors that are difficult to measure accurately. At the same time, the clinical course, symptomatology, and pathological mechanism of PD are different in different patients, and a variety of molecular mechanisms coexist. Neuroimaging is likely to play a crucial part in future studies to identify the hazard of PD. Prospective cohort studies are currently attempting to evaluate the sensitivity, specificity, and predictive value of all kinds of risk markers. Biomarkers are made use to evaluate the risk and progression of the disease or to rise early diagnosis up. You have to find markers that can be used to find precursors. Variations in the intestinal microbiota of PD have been the concentration upon research in the last several years. We need to find out which gut microbiota is involved in PD and which drugs are effective in treating it. PD is connected with a series of pathophysiological processes, involving neuronal vulnerability, neural network alterations, α‐Synuclein aggregation, mitochondrial dysfunction, glutamate toxicity, apoptosis theory, immunoinflammatory mechanism (Figure 6). In summary, the pathogenic mechanisms of PD have not been completely elucidated, large amounts of theories and agreed‐upon conclusions are still coexisting. The complicacy of a few intertwined pathways and the heterogeneousness in clinical types will require specific therapy. In summary, the pathogenesis of PD has not been fully elucidated, and large amounts of theories and agreed‐upon conclusion. The complexity of these intertwined pathways and the heterogeneity in clinical phenotypes will require a targeted approach for therapy. But the radical cure for primary PD has not been discovered so far. l‐Dopa is one of the most common drugs used in clinical practice. It is the improvement of patients' symptoms in a short time; however, side effects increase, including cough, nausea, and so forth over time. Thus, in recent years, clinical trials have often been conducted to enhance the efficacy of l‐Dopa by altering its dose or combining it with other drugs. What's more, surgery is one of the main treatments for patients with PD; however, complications, such as epilepsy and sleep disorders are not negligible. DBS is more effective than medicine therapy alone in reducing the side effects of PD. The location of DBS and the high or low frequency can offer various results to this therapy. DBS has played a neuroregulatory role in clinical application, but the exact mechanism is still unclear. Any treatment method is limited, and DBS is no exception. In most cases, only a few targets are selected for DBS treatment, and most of them are not fundamentally different, so it is unrealistic to solve various symptoms. It may also cause surgical complications, resulting in venous reflux disorders, the formation of cerebral hematoma, secondary to multiple organ failure. Louie pathology appeared within transplanted neurons more than 10 years after surgery. Consensus is consistent and shows signs of degradation (loss). Cell transplantation is considered a promising method of brain research for repairing PD, where some patients have been found to have uncontrollable graft‐induced responses. However, nerves are expressed only in relatively stable cells, with few cells around the injection path, limiting their potential for nerve repair. In addition, α‐Synuclein and immunotherapy are also experimental therapies for PD. Gene therapy aimed at PD is a novel method with potential, while a series of deficiencies exist in operation technology. Currently, α‐Synuclein is considered to be closely related to the related dysfunction and pathogenesis of PD, which is a kind of soluble protein, and expressed in the central nervous system in the presynaptic and perinuclear regions. There is an inconsistent relationship between α‐Synuclein gene polymorphism and cognitive function, and α‐Synuclein targeting methods mainly concentrate on the extracellular α‐Synuclein binding sites. Specific exercise and technology programs of computers related to PD are novel treatments for patients with PD. Though these are still in the experimental stage, if further research continues, these measures might become clinical treatments one day. In conclusion, these advances show that the prospect of PD treatment is bright.

## CONFLICT OF INTERESTS

The authors declare that there are no conflict of interests.

## ETHICS STATEMENT

Ethics statement for the article is not applicable.

## AUTHOR CONTRIBUTIONS

Ting‐Ting Yang contributed the central idea and wrote the initial draft of the paper. Yu‐Cong Liu, Jing Li, Hui‐Chan Xu, and Shun‐Lian Li contributed to refining the ideas, carrying out additional analyses. Ting‐Hua Wang and Liu‐Lin Xiong reviewed and edited this paper.

7

## Data Availability

Data sharing is not applicable to this article
